# Consolidation Tumor Ratio Combined With Pathological Features Could Predict Status of Lymph Nodes of Early-Stage Lung Adenocarcinoma

**DOI:** 10.3389/fonc.2021.749643

**Published:** 2022-01-14

**Authors:** Liang Zhao, Guangyu Bai, Ying Ji, Yue Peng, Ruochuan Zang, Shugeng Gao

**Affiliations:** ^1^ Department of Thoracic Surgery, National Cancer Center/National Clinical Research Center for Cancer/Cancer Hospital, Chinese Academy of Medical Sciences and Peking Union Medical College, Beijing, China; ^2^ Department of Thoracic Surgery, Beijing Chaoyang Hospital, Capital Medical University, Beijing, China

**Keywords:** lung, part-solid nodules, adenocarcinoma, lymph node metastasis, nomogram

## Abstract

**Introduction:**

Stage IA lung adenocarcinoma manifested as part-solid nodules (PSNs), has attracted immense attention owing to its unique characteristics and the definition of its invasiveness remains unclear. We sought to develop a nomogram for predicting the status of lymph nodes of this kind of nodules.

**Methods:**

A total of 2,504 patients between September 2018 to October 2020 with part-solid nodules in our center were reviewed. Their histopathological features were extracted from paraffin sections, whereas frozen sections were reviewed to confirm the consistency of frozen sections and paraffin sections. Univariate and multivariate logistic regression analyses and Akaike information criterion (AIC) variable selection were performed to assess the risk factors of lymph node metastasis and construct the nomogram. The nomogram was subjected to bootstrap internal validation and external validation. The concordance index (C-index) was applied to evaluate the predictive accuracy and discriminative ability.

**Results:**

We enrolled 215 and 161 eligible patients in the training cohort and validation cohort, respectively. The sensitivity between frozen and paraffin sections on the presence of micropapillary/solid subtype was 78.4%. Multivariable analysis demonstrated that MVI, the presence of micropapillary/solid subtype, and CTR >0.61 were independently associated with lymph node metastasis (*p* < 0.01). Five risk factors were integrated into the nomogram. The nomogram demonstrated good accuracy in estimating the risk of lymph node metastasis, with a C-index of 0.945 (95% CI: 0.916–0.974) in the training cohort and a C-index of 0.975 (95% CI: 0.954–0.995) in the validation cohort. The model’s calibration was excellent in both cohorts.

**Conclusion:**

The nomogram established showed excellent discrimination and calibration and could predict the status of lymph nodes for patients with ≤3 cm PSNs. Also, this prediction model has the prediction potential before the end of surgery.

## Introduction

Early-stage lung adenocarcinoma manifested as part-solid nodules (PSNs) has attracted much attention owing to its unique characteristics. However, no consensus has been reached on its invasiveness, and the incidence of lymph node metastasis is elusive. Emerging evidence shows that lymph node metastasis is a risk factor of poor prognosis for patients with early-stage lung adenocarcinoma (1), and various factors (2–4) are associated with the incidence of positive lymph nodes.

Consolidation tumor ratio (CTR) of part-solid nodules, for instance, is one of the critical factors associated with lymph node metastasis. Kenji et al. (5) claimed that CTR could predict invasiveness of part-solid nodules (≤2 cm), whereas nodules with CTR <0.25 were not associated with positive lymph nodes (5). Two years later, their colleagues reported updated findings, whereby they adjusted the nodule size and the cutoff value to 3 cm and 0.5, respectively (6). In this context, our Japanese colleges conducted a series of clinical trials to explore the standard surgical procedures for early-stage lung cancers (7). A systematic review published in 2017 demonstrated that patients with CTR >80% were more prone to lymph node metastasis, and their disease-free survival (DFS) and overall survival (OS) were significantly lower than that of patients with CTR ≤80% (4). Therefore, they suggested a clear definition of the upper limit of the solid component of PSNs.

Other than CTR, some clinical parameters have widely been applied to predict lymph node metastasis, including CEA (3), tumor size (3), and Standardized Uptake Value max (SUVmax) (2). However, pathological features associated with histological subtypes are hardly utilized to predict the status of lymph nodes owing to their hysteresis. There is evidence that micropapillary or solid predominant adenocarcinoma is characterized by the worst prognosis. Also, the presence of minor micropapillary or solid histological subtypes is an independent risk factor of lymph node metastasis and poor prognosis in patients with lung adenocarcinomas (8–10). Some researchers have claimed that frozen sections are a crucial indicator to guide the resection method (11), and it is feasible to report histological subtypes and other pathological features during operation (12, 13).

Here, we reviewed a series of patients with PSNs and integrated their clinical and pathological characteristics to construct a nomogram. What is more, we also verified the consistency between FS and paraffin sections, aiming to enable the nomogram to predict the incidence of lymph node metastasis before the end of surgery, which may provide a calculation method for surgeons to make intraoperative decisions.

## Materials and Methods

### Patients

We retrospectively studied patients with PSNs who had undergone lobectomy and systemic lymph node dissection from our prospective database (Department of Thoracic Surgery, National Cancer Center database) between October 2018 and September 2020. Patient informed consent was waived because of the retrospective nature of the study. Patients received no financial compensation.

Eligible patients enrolled between October 2018 and September 2019 constituted a training cohort for developing the nomogram, whereas those enrolled between October 2019 and September 2020 formed the validation cohort. The inclusion criteria were as follows: (1) patients who underwent lobectomy and systemic lymph node dissection; (2) T1a-cN0 clinical stage; (3) primary adenocarcinoma as the pathological type; and (4) lung nodules manifested as part-solid nodules on computer tomography (CT) images. The exclusion criteria were as follows: (1) patients with noninvasive pathological types, including adenocarcinoma *in situ* (AIS) and minimally invasive adenocarcinoma (MIA); (2) subjects who received any preoperative anticancer treatments; and (3) patients with a history of other malignant tumors.

The ethics committee of the National Cancer Center/Cancer Hospital, Chinese Academy of Medical Sciences, and Peking Union Medical College approved this study (approval number: 21/301-2972).

### Radiological Evaluation

Each patient from the two cohorts underwent a high-resolution CT scan 2–4 weeks before the operation. The CT layer thickness was 1.25 mm. Lung images were analyzed at a window level of −500 to −700 H and a window width of 1,000 to 2,000 H. Tumor size (defined as the maximum diameter of the lesion on CT scan) was measured in the lung window. The consolidation component was defined as an area of increased opacification that completely obscured the underlying vascular markings (5). Ground glass opacity (GGO) was defined as an area of a slight, homogenous increase in density that did not obscure the underlying vascular markings (14). Two experienced senior radiologists, blinded to pathological diagnosis, re-evaluated CT images of all patients independently.

### Histological Evaluation

Tissue sections were subjected to hematoxylin and eosin staining and elastic fiber staining to evaluate the following pathological factors: grade of differentiation; the maximum diameter of the primary tumor and central fibrosis; pleural involvement; microscopic vessel invasion (MVI); and histological subtypes. Pathologic diagnosis was according to the 2015 World Health Organization (WHO) classification of lung cancer. The staging standard followed the 8th edition of the Union for International Cancer Control/American Joint Committee on Cancer (UICC/AJCC) TNM staging for NSCLC (15, 16). The degree of tumor histological differentiation was classified into three grades: (I) well-differentiated; (II) moderately differentiated; and (III) poorly differentiated. The five histological subtypes (lepidic, acinar, papillary, micropapillary, and solid) were recorded semiquantitatively in 5% increments (17). We defined “the presence of micropapillary or solid subtypes” as the percentage of micropapillary or solid subtypes is equal to or over 5%.

Two senior pathologists reevaluated the frozen sections. Micropapillary or solid subtypes between frozen sections and paraffined sections were compared. Measurement of consistency between the types kinds of sections was taken. We also tried to reevaluate the MVI in the frozen sections though it seems a little complicated.

### Statistical Analysis

Continuous variables were compared using an unpaired, 2-tailed *t*-test or Mann-Whitney *U* test and presented as median (interquartile range). Categorical variables were compared using the *χ* ^2^ test or Fisher’s exact test. To analyze the risk factors of lymph node metastasis, the significance of each variable in the training cohort was assessed by univariate logistic regression analysis. Backward stepwise selection with the Akaike information criterion (AIC) was employed to identify variables for multivariate logistic regression models and nomograms. Odds ratios (ORs) were presented with their 95% CIs. The “rms” package of R version 4.0 (http://www.r-project.org/) was employed to construct a nomogram, based on multivariate logistic regression analysis results. Each regression coefficient in multivariate logistic regression was proportionally converted to a 0- to 100-point scale. The points were added across variables to derive total points, which were converted to predicted probabilities. The predictive performance of the nomogram was measured by concordance index (C-index) and calibration with 2,000 bootstrap samples to reduce the overfit bias. In all analyses, *p* < 0.05 denoted statistical significance. All analyses were performed in SPSS version 25 and R version 4.0.

## Results

### Clinicopathologic Characteristics of Patients

**Figure 1:**
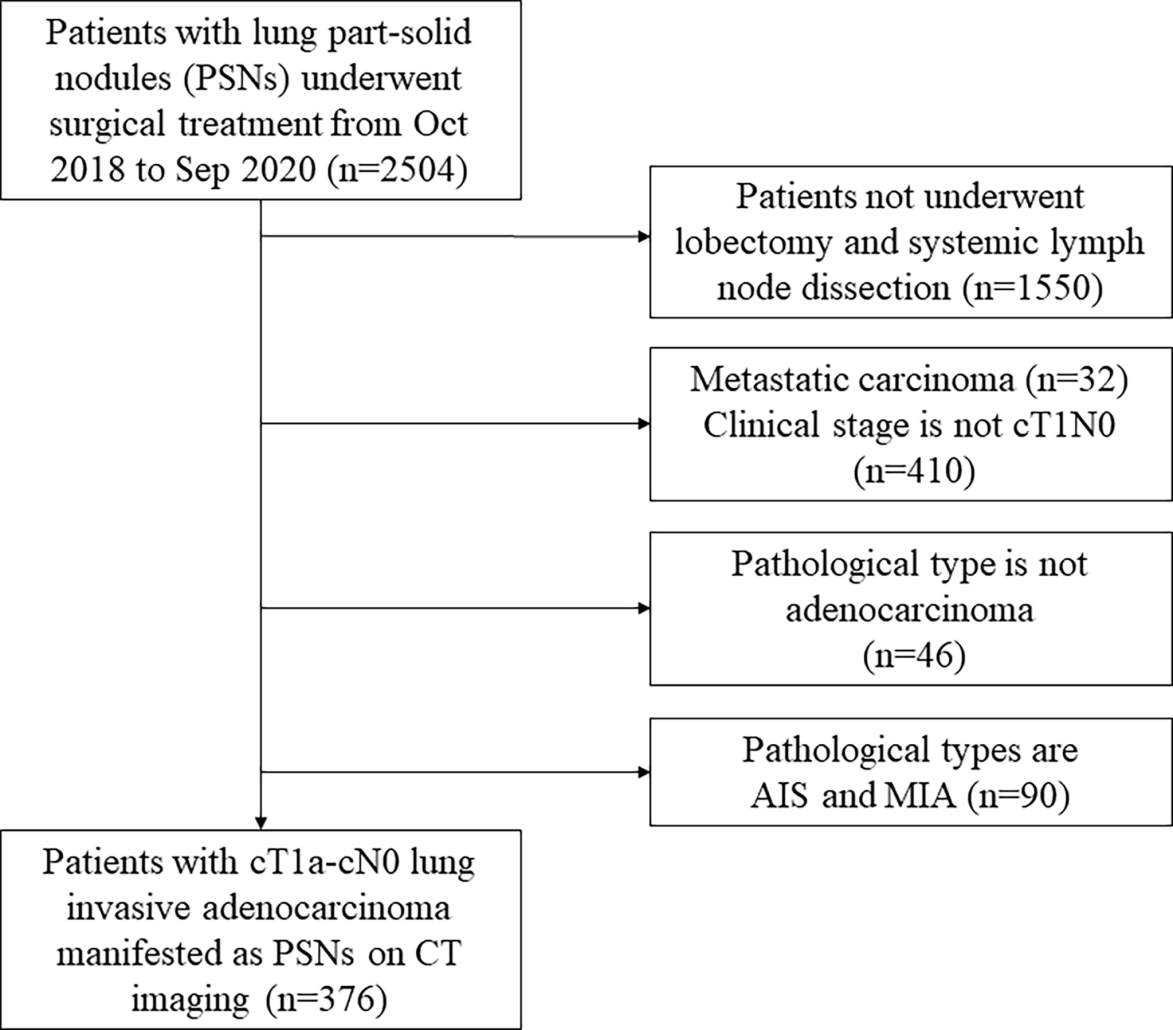
The flow diagram of the patient selection. PSN, part-solid nodule; AIS, adenocarcinoma in situ; MIA, microinvasive adenocarcinoma.

We studied 376 consecutive patients with cStageT1a-cN0 lung invasive adenocarcinoma. All lung nodules manifested as part-solid nodules and were radically resected (**Figure 1**). Notably, 215 patients from October 2018 to September 2019 were enrolled in a training cohort, while the other 161 patients from October 2019 to September 2020 were included in the validation cohort.

**Figure 2:**
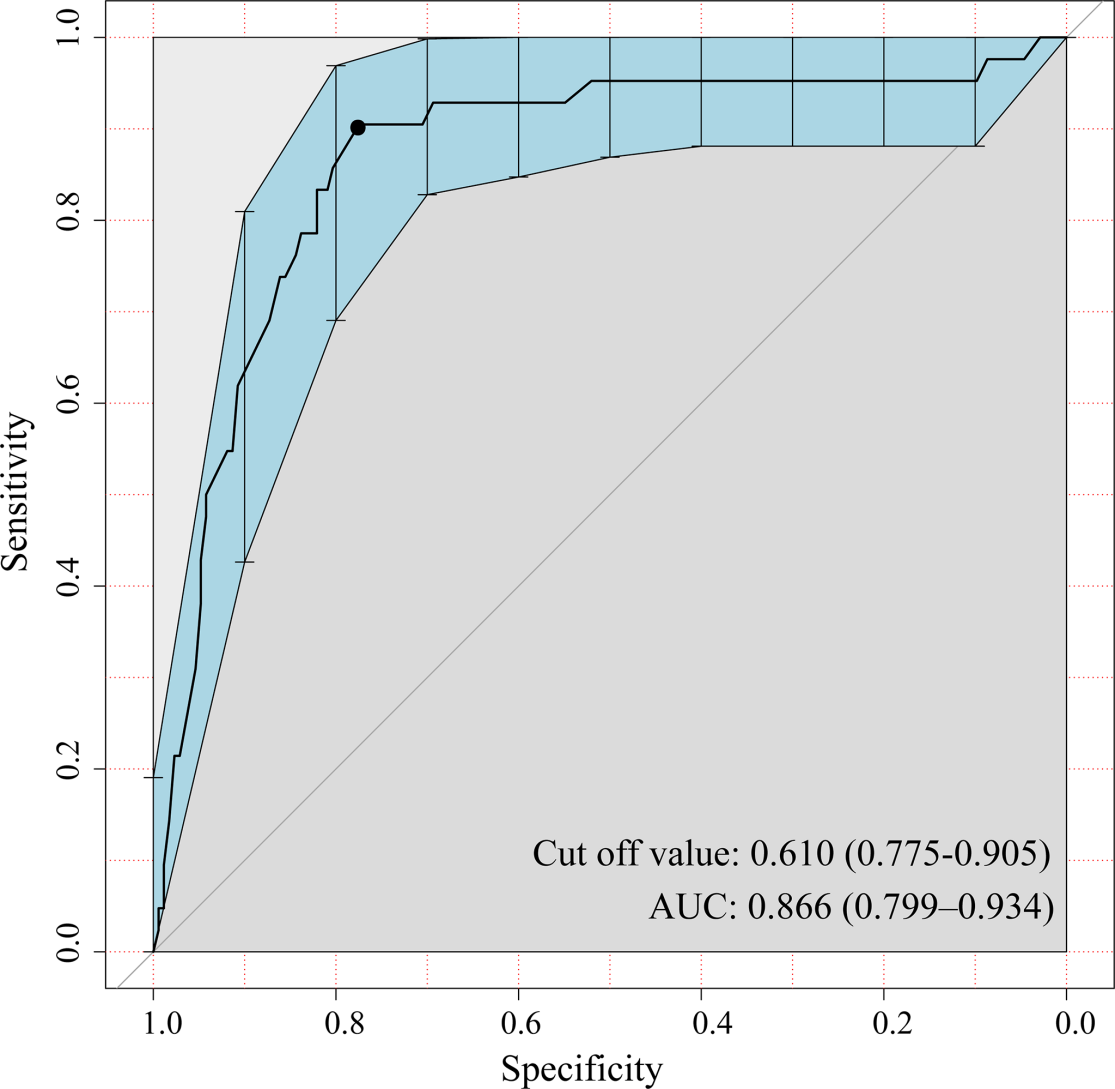
A representative receiver operating characteristic (ROC) curve displayed the classification performance of consolidation tumor ratio (CTR). The cutoff value is 0.61, which means PSNs are more prone to have positive lymph nodes when CTR >0.61. The area under curve (AUC) is 0.866; 95% confidence interval (CI) is shown in blue shade (0.799, 0.934).

**Table 1** outlines the clinicopathologic characteristics of the patients. The positive ratio of lymph nodes was 19.5% (42/215) and 26.1% (42/161) in the two cohorts, respectively. The consistency between frozen sections and paraffin sections is shown in **Tables 2** and **3**. The sensitivity and specificity of the presence of micropapillary/solid subtype was 78.4% (80/102) and 97.4% (267/274), respectively. For MVI, the sensitivity and specificity were 51.3% (20/39) and 99.4% (335/337). The negative predictive value of the presence of micropapillary/solid subtype and MVI was 92.4% and 94.6%, respectively. The receiver operating characteristic (ROC) curve showed a cutoff value of CTR, at 0.61, whereas the sensitivity and specificity of positive lymph nodes were 91.1% and 78.8%, respectively (**Figure 2**). The area under curve (AUC) was 0.87 (95% CI: 0.80, 0.93) with *p* < 0.01.

**Table 1:** Clinical and pathological characteristics of patients.

Characteristics	Median (range) or number (%)		
	Training cohort	Validation cohort	Total
Age	58 (53–63)	61 (56–67)	59 (53–64)
Gender			
Female	150 (69.8)	101 (62.7)	251 (66.8)
Male	65 (30.2)	60 (37.3)	125 (33.2)
cStage			
cT1N0	215 (100)	161 (100)	376 (100)
cT1N1-2	0 (0)	0 (0)	0 (0)
pStage			
pT1N0	173 (80.5)	119 (73.9)	292 (77.7)
pT1N1-2	42 (19.5)	42 (26.1)	84 (22.3)
Differentiation			
High	67 (31.2)	60 (37.3)	127 (33.8)
Median	105 (48.8)	48 (29.8)	153 (40.7)
Low	43 (20)	53 (32.9)	96 (25.5)
Micropapillary/solid subtype			
Absent	156 (72.6)	118 (73.3)	274 (72.9)
Present	59 (27.4)	43 (26.7)	102 (27.1)
MVI			
Yes	24 (11.2)	15 (9.3)	39 (10.4)
No	191 (88.8)	146 (90.7)	337 (89.6)
Vis PI			
Yes	1 (0.4)	3 (1.9)	4 (1.1)
No	214 (99.6)	158 (98.1)	372 (98.9)
Nerve invasion			
Yes	2 (0.9)	1 (0.6)	3 (0.8)
No	213 (99.1)	160 (99.4)	373 (99.2)
Necrosis			
Yes	3 (1.4)	2 (1.2)	5 (1.3)
No	212 (98.6)	159 (98.8)	371 (98.7)
Tumor size (cm)	1.98 (1.53–2.48)	1.80 (1.40–2.30)	1.93 (1.50–2.40)
Consolidation component length (cm)	0.86 (0.49–1.53)	0.92 (0.61–1.32)	0.87 (0.50–1.47)
CTR			
<0.61	138 (64.2)	112 (69.6)	250 (66.5)
>0.61	77 (35.8)	49 (30.4)	126 (33.5)

MVI, microscopic vessel invasion; VisPI, visceral pleural infiltration; CTR, consolidation tumor ratio.

**Table 2:** The consistency of the presence of micropapillary/solid subtype between frozen sections and paraffin sections.

Frozen sections	Paraffin sections		Total
	Positive	Negative	
Positive	80	7	87
Negative	22	267	289
Total	102	274	376

**Table 3:** The consistency of MVI between frozen sections and paraffin sections.

Frozen sections	Paraffin sections		Total
	Positive	Negative	
Positive	20	2	22
Negative	19	335	354
Total	39	337	376

MVI, microscopic vessel invasion.

### Independent Risk Factors for Lymph Node Metastasis

The results of univariate and multivariate logistic regression are presented in **Table 4**. Univariate analysis implicated low histological differentiation, the presence of micropapillary/solid subtype, microscopic vascular invasion (MVI), tumor size, consolidation component size, and CTR >0.61 are risk factors on lymph node metastasis. After AIC selection of variables, multivariate analysis with an odds ratio (95% CI) showed that MVI (22.29 [4.04, 122.92]), the presence of micropapillary/solid subtype (5.09 [1.80, 14.40]), and CTR >0.61(12.49 [2.63, 59.40]) were independently associated with lymph node metastasis (*p* < 0.01), while the age was slightly significant (*p* = 0.05).

**Table 4:** Univariate and multivariate analyses of lymph node metastasis.

Variables	Univariate analysis			Multivariate analysis (before AIC)			Multivariate analysis (after AIC)		
	OR	*p*-value	95% CI	OR	*p*-value	95% CI	OR	*p*-value	95% CI
Age	1.01	0.56	0.97, 1.06				1.07	0.05	1.00, 1.14
Gender (reference: female)	1.2	0.61	0.60, 2.42						
Histological differentiation (reference: high)									
Median	2.03	0.24	0.63, 6.59	0.29	0.13	0.06,1.43			
Low	32.63	<0.01^*^	9.88, 107.79	1.61	0.62	0.25, 10.51			
Micropapillary/solid subtype(reference: no)	15.23	<0.01^*^	6.97, 33.28	1.84	0.39	0.46, 7.36	5.09	<0.01^*^	1.80, 14.40
MVI (reference: no)	33.2	<0.01^*^	10.48, 105.16	24.03	<0.01^*^	4.21, 137.26	22.29	<0.01*	4.04, 122.92
Tumor size	2.03	<0.01^*^	1.30, 3.16	0.70	0.65	0.15, 3.24			
Consolidation component length	6.61	<0.01^*^	3.69, 11.84	2.91	0.25	0.47, 18.09	1.63	0.31	0.64, 4.15
CTR(reference: <0.61)	38.15	<0.01^*^	12.82, 113.55	7.88	0.47	1.03,60.12	12.49	<0.01*	2.63, 59.40

OR, odds ratio; MVI, microscopic vessel invasion; Vis PI, visceral pleura invasion. *P value < 0.05.

### Nomograms and Model Performance

**Figure 3:**
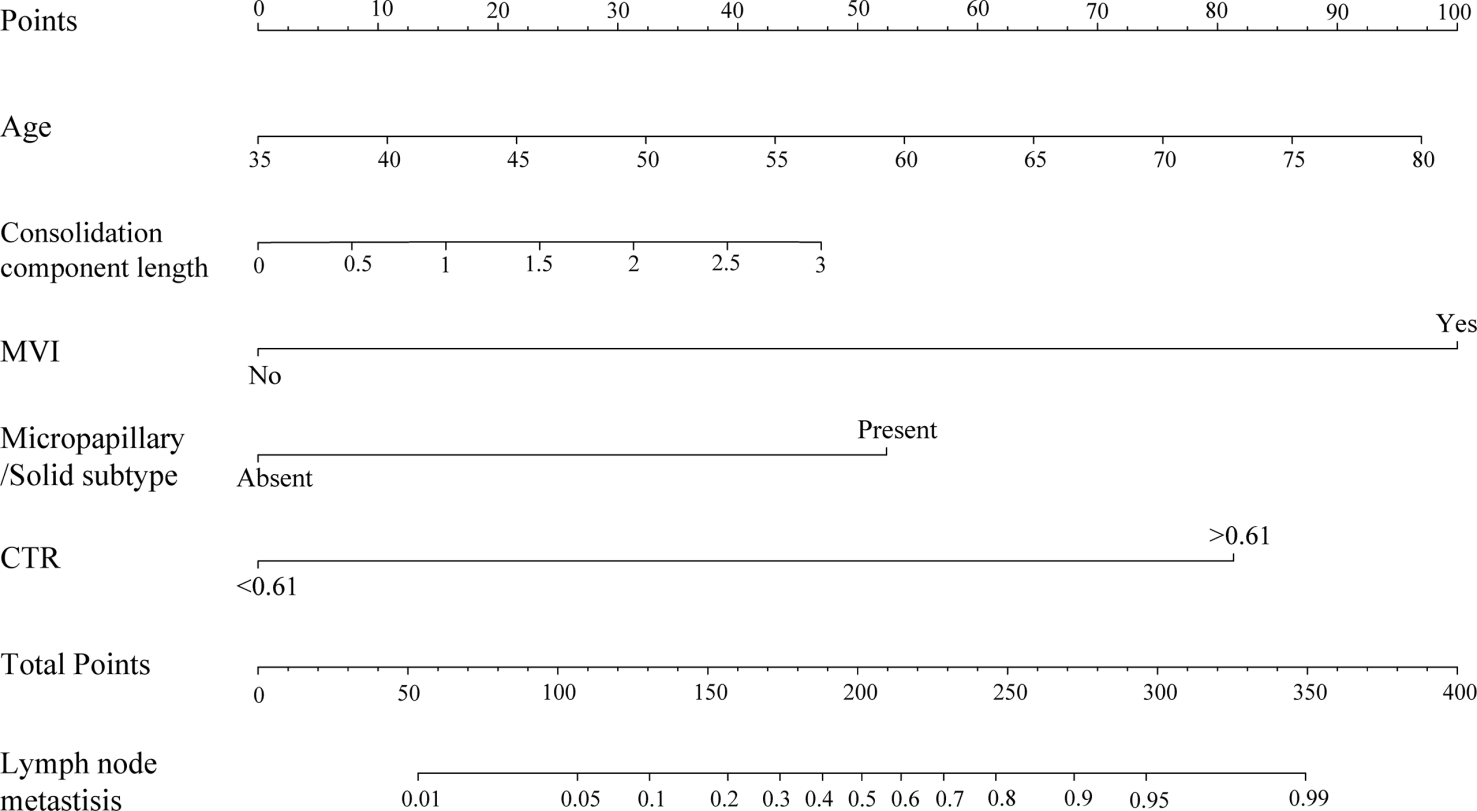
Nomogram for the prediction of lymph node metastasis of patients with lung adenocarcinoma manifested as PSNs. To use the nomogram, find the position of each variable on the corresponding axis, draw a line to the points axis for the number of points, add the points from all of the variables, and draw a line from the total points axis to determine the probabilities of lymph node metastasis at the lower line of the nomogram.

**Figure 4:**
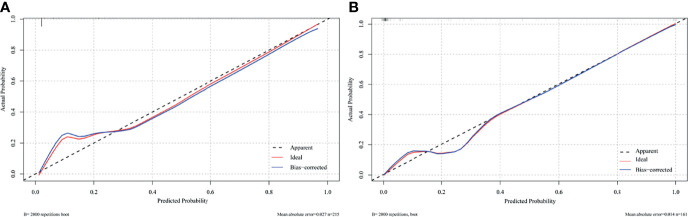
Calibration plot comparing predicted and actual probability of lymph node metastasis in **(A)** training cohort (*n* = 215) and **(B)** validation cohort (*n* = 161).

The nomogram was constructed based on the AIC selection and clinical characteristics (AIC = 114.23). MVI, CTR >0.61, age, consolidation component length, and the presence of micropapillary/solid subtype were integrated into the nomogram (**Figure 3**). The *p*-value of the Hosmer-Lemeshow test was 0.83, indicating an acceptable fit of our model. The model was internally validated using the bootstrap validation method, whereas external validation was performed in the validation cohort. The nomogram demonstrated good accuracy in estimating the risk of lymph node metastasis, with a C-index of 0.945 (95% CI, 0.916–0.974). Furthermore, calibration plots showed good agreement on the presence of lymph node metastasis between the estimation and histopathological examination (**Figure 4A**). The total scores of each patient in the validation cohort were calculated using the nomogram, and the total points in this cohort were integrated into logistic regression as a factor. Finally, the C-index (0.975, 95% CI: 0.954–0.995)) and the calibration curve (**Figure 4B**) of the validation cohort were derived.

## Discussion

In this retrospective study, we developed a nomogram to predict the incidence of lymph node metastasis. MVI, CTR >0.61, and the presence of micropapillary/solid subtype were revealed as independent risk factors of lymph node metastasis. The consistency of the presence of micropapillary/solid subtype between paraffin sections and frozen sections was excellent, while the sensitivity of MVI seems inappropriate. Our nomogram demonstrated excellent discrimination and calibration, and it first considered the use of frozen sections. Therefore, the nomogram might have prediction potential for lymph node status before the end of surgery and guide surgeons to develop lymph node dissection strategy.

The incidence of lymph node metastasis of lung nodules was widely studied before (2, 3), and CTR had already been used to predict lymph node metastasis, especially in Asia (5, 6). Chen et al. claimed that CTR was not inferior to primary tumor SUVmax considering the predictive power for lymphatic metastasis preoperatively in lung cancer patients with a GGO component (18). Also, Japan carried out a series of clinical trials based on the CTR (7). They considered lung nodules as imaging noninvasive when their CTR <0.5, whereas the sensitivity and specificity are 96.4% and 30.4%, respectively (6). Inconsistent with their results, we found 0.61 as a cutoff value of CTR and the sensitivity and specificity of 91.1% and 78.8%, respectively. Compared with them, our higher specificity could help surgeons avoid extended lymph node dissection, which might reduce the operation time and the frequency of postoperative complications (19). Also, if there were a low incidence of lymph node metastasis, lobe-specific lymph node dissection would be a relatively safe surgery option (20). Moreover, other cutoff values of CTR [such as 0.62 (18), 0.86 (21), and 0.8 (4)] were also reported to be associated with positive lymph nodes, demonstrating CTR may not have a precise cutoff value. We presume that several reasons may contribute to the differences in CTR cutoff values: (1) it is inaccurate to establish the relationship between CTR and lymph node metastasis separately, and other imaging features should be introduced as variables. For instance, current evidence indicates that imaging omics combined with deep learning are valuable approaches in predicting the malignancy of lung nodules (22); (2) the relationship between CTR and lymph node metastasis is not a simple linear relationship, and there may be some complicated mathematical relationship between them. Taken together, we cannot give a precise suggestion as to what value the specific CTR should take but rather find a reasonable cutoff value of CTR under the existing conditions. The application of this indicator is still a subject worthy of study in the future.

In this article, we also found MVI and the presence of micropapillary/solid subtype are significant risk factors on lymph node metastasis. MVI had been reported as an independent risk factor for OS and DFS (23, 24). Neri et al. reported a correlation of MVI with epithelial-mesenchymal transition (EMT) and cancer stemness (CS), which may contribute to poor outcomes (25). The inner molecular mechanism of MVI is yet to be explored. Moreover, the presence of micropapillary/solid subtype had also been studied before; for instance, the International Association for the Study of Lung Cancer (IASLC) had brought them into a new pathological grading system because patients were characterized by poor DFS and OS (26). Elsewhere, Watanabe et al. reported that the micropapillary subtype was associated with short recurrence time (10), and Qian et al. claimed that patients with these particular subtypes would benefit from adjuvant chemotherapy postoperation (27).

It is worth mentioning that frozen sections could assist in diagnosing the presence of micropapillary/solid subtype whose sensitivity and negative predictive values were 78.4% and 92.4%, respectively. However, the diagnosis of MVI lacked corresponding studies. Although the sensitivity of MVI is only 51.3% in our study, this value has been somewhat too high in clinical practice. This may affect the clinical application of our nomogram, but one of our purposes is to explore the possibility of applying the model before the end of surgery. Jiang et al. showed that intraoperative frozen sections could potentially change the surgical resection strategy by influencing postoperative OS and DFS whose sensitivity of the presence of micropapillary is up to 74.2% (12). They believe that the misdiagnosis was caused by sampling error (lack of tissue) and interpretation error (other atypical micropapillary subtypes). Here, we obtained a higher sensitivity of frozen sections and firstly used frozen sections to diagnose MVI. It gives us hope that in the future, with the development of the diagnostic technology of pathology, frozen sections could serve as a reference for resection strategy. Currently, the 101st American Association for Thoracic Surgery (AATS) disclosed a summary of the results of the JCOG0802 trial, which stated that for peripheral lung cancer (≤2 cm, CTR >0.5), the prognosis of segmentectomy is better than that of lobectomy. However, the subgroup analysis results of histopathological subtypes are unknown. Whether segmentectomy is sufficient for lung adenocarcinoma containing micropapillary/solid components and MVI remains a research gap that needs urgent exploration in the future.

Based on previous evidence, many prediction models were focused on predicting the lymph node status of PSNs, but a majority only adopted imaging features as indicators (28). Herein, the presently established nomogram has five variables, and it firstly integrated both imaging features and histological features for a more accurate predictive function. Furthermore, the discrimination and calibration potential of our nomogram are excellent, with a great C-index, demonstrating that it is of great promise in clinical practice. The external validation also showed excellent discrimination and calibration, implying that the model has good extrapolation ability. Nevertheless, there are still some factors we did not involve, including CEA level, SUVmax, and so on, for the lack of data (not all early-stage lung cancer patients need to take PET-CT and CEA examination). Current data showed that the combined application of multiomics, including liquid biopsy, proteomics, and metabolomics, has widened in predicting the malignancy of small pulmonary nodules (29). We believe that a prospective cohort will be established in the future to develop a more complete model to predict the incidence of lymph node metastasis of early-stage lung adenocarcinoma.

Our study had some limitations. First, this analysis was based on data from a single institution; it would be imperative to validate the results from other centers. Second, the number of training patients is small, which may have introduced selection bias. Third, because the model was based on clinicopathologic data, it may lack other indicators, and their effect or potential incorporation in the nomograms could not be assessed.

## Conclusions

In summary, for PSNs diagnosed as early-stage lung adenocarcinoma, MVI, the presence of micropapillary/solid subtype, and CTR >0.61 are significant risk factors associated with lymph node metastasis. A nomogram containing five variables was established, and in the future, based on this nomogram, surgeons might predict lymph node status before the ending of surgery.

## Data Availability Statement

The raw data supporting the conclusions of this article will be made available by the authors, without undue reservation.

## Ethics Statement

The studies involving human participants were reviewed and approved by the National Cancer Center/Cancer Hospital, Chinese Academy of Medical Sciences, and Peking Union Medical College approved this study. Written informed consent for participation was not required for this study in accordance with the national legislation and the institutional requirements.

## Author Contributions

SG, LZ, and GB contributed to the conception and design of the study. GB organized the database. GB and YJ performed the statistical analysis. GB wrote the first draft of the manuscript. LZ, YJ, YP, and RZ wrote sections of the manuscript. All authors contributed to manuscript revision, read, and approved the submitted version.

## Funding

This work was supported by the Beijing Hope Marathon Special Fund of Cancer Foundation of China (LC2018A02).

## Conflict of Interest

The authors declare that the research was conducted in the absence of any commercial or financial relationships that could be construed as a potential conflict of interest.

## Publisher’s Note

All claims expressed in this article are solely those of the authors and do not necessarily represent those of their affiliated organizations, or those of the publisher, the editors and the reviewers. Any product that may be evaluated in this article, or claim that may be made by its manufacturer, is not guaranteed or endorsed by the publisher.
